# Agent

**Published:** 1857-04

**Authors:** 


*Agent.*

Mr. John B. White, of Aurora, has been duly authorized to act as Agent for this *Journal*, both for soliciting new subscribers and collecting money from those who are already in arrears. But many of those who are indebted to us for one or more years' subscription, live off from the railroad lines, and so scattered that it is very inconvenient for an agent to visit them: we hope all such will speedily remit to us by mail, without waiting for an agent to make personal application to them.

